# A Comparison of the Use of Handheld KardiaMobile ECG Devices With 12-Lead ECGs in an Older Adult Psychiatric Setting

**DOI:** 10.1192/bjo.2024.133

**Published:** 2024-08-01

**Authors:** Ben Cross, Jane Leadbetter, Anna Richman

**Affiliations:** Mersey Care NHS Foundation Trust, Liverpool, United Kingdom

## Abstract

**Aims:**

To establish the usability and tolerability, as well as accuracy of measurements of a handheld KardiaMobile ECG device in an inpatient older adult dementia ward.

**Methods:**

Between February 2023 and April 2023, KardiaMobile ECGs and 12-lead ECGs were taken for patients admitted within a dementia ward in Liverpool. The standard 12-lead ECGs were analysed as per current practice, by Broomwell Health Watch. The KardiaMobile ECGs were read manually, by two independent raters, for heart rate and QTc. The user-rated tolerability was measured out of 5, 5 being the most tolerable, and was measured for both KardiaMobile and 12-lead ECGs, allowing comparison. The QTc and heart rate were calculated for both methods, and then compared. QTc was calculated using Bazett's formula.

**Results:**

13 inpatients had a 12-lead ECG, and a KardiaMobile ECG performed. Both were tolerated by all patients, except one who tolerated neither, leaving 12 ECGs for comparison. KardiaMobile ECGs were quicker to obtain, more well tolerated, and easy to use. However, manual calculation of QTc, versus expert and computer analysis for 12-lead ECGs, led to some variability between QTc measurements. Inter-rater reliability between raters for the KardiaMobile QTc was poor, however, when both were combined, correlation with 12-lead ECG QTc was moderate. KardiaMobile ECGs were harder to obtain in those with tremors, and the lack of computerised readings made interpretation more difficult. 12-lead ECGs also offer reassurance in the form of a fully interpreted, more detailed ECG.

**Conclusion:**

KardiaMobile devices are faster to use and as/more tolerable in a dementia ward setting than 12-lead ECGs. The ECG trace is fed back instantly to the mobile device, however, automatic interpretation is limited and QTc calculation relies on the operator. Visual inspection of QTc can be difficult, and unreliable. However, the combination of two different raters led to more reliable results. The device has potential for use in this setting, however, an increase in automatic interpretation, or interpretation by a third party such as with Broomwell Health Watch, would increase its usability.

